# In vitro and in vivo studies of plant-produced Atezolizumab as a potential immunotherapeutic antibody

**DOI:** 10.1038/s41598-023-41510-w

**Published:** 2023-08-29

**Authors:** Kaewta Rattanapisit, Christine Joy I. Bulaon, Richard Strasser, Hongyan Sun, Waranyoo Phoolcharoen

**Affiliations:** 1 Baiya Phytopharm Co., Ltd., Bangkok, Thailand; 2 Center of Excellence in Plant-Produced Pharmaceuticals, Chulalongkorn University, Bangkok, 10330 Thailand; 3 Department of Pharmacognosy and Pharmaceutical Botany, Faculty of Pharmaceutical Sciences, Chulalongkorn University, Bangkok, 10330 Thailand; 4 Department of Applied Genetics and Cell Biology, University of Natural Resources and Life Sciences, Muthgasse 18, 1190 Vienna, Austria; 5 GemPharmatech Co., Ltd, Nanjing, China

**Keywords:** Biotechnology, Immunology, Molecular biology, Plant sciences

## Abstract

Immune checkpoint inhibitors are a well-known class of immunotherapeutic drugs that have been used for effective treatment of several cancers. Atezolizumab (Tecentriq) was the first antibody to target immune checkpoint PD-L1 and is now among the most commonly used anticancer therapies. However, this anti-PD-L1 antibody is produced in mammalian cells with high manufacturing costs, limiting cancer patients’ access to the antibody treatment. Plant expression system is another platform that can be utilized, as they can synthesize complex glycoproteins, are rapidly scalable, and relatively cost-efficient. Herein, Atezolizumab was transiently produced in *Nicotiana benthamiana* and demonstrated high expression level within 4–6 days post-infiltration. After purification by affinity chromatography, the purified plant-produced Atezolizumab was compared to Tecentriq and showed the absence of glycosylation. Furthermore, the plant-produced Atezolizumab could bind to PD-L1 with comparable affinity to Tecentriq in ELISA. The tumor growth inhibitory activity of plant-produced Atezolizumab in mice was also found to be similar to that of Tecentriq. These findings confirm the plant’s capability to serve as an efficient production platform for immunotherapeutic antibodies and suggest that it could be used to alleviate the cost of existing anticancer products.

Cancer is a disease that occurs when tumor cells grow uncontrollably and spread to other parts of the body. Since then, it has become one of the leading causes of death in humans, with the greatest impact in developing countries^1,2^. Cancer is treated using a variety of methods, including surgery, chemotherapy, radiation therapy, and immunotherapy^3^. Immunotherapeutic treatments assist the immune system in combating cancer. Immune checkpoint inhibitors (ICIs), adoptive cell transfer therapy, and cancer vaccines, are among the main immunotherapies used to treat cancer^4^.

ICIs are monoclonal antibodies (mAbs) that target and block the inhibitory immune checkpoints such as, but not limited to, PD-1, PD-L1 and CTLA-4^5–7^. The binding of PD-1 on T cells and PD-L1 on cancer cells, for example, inhibits T cell killing of cancer cells. When PD-1/PD-L1 binding is blocked with an ICI, T cells can kill cancer cells, taking advantage of body’s own immune cells to attack tumor cells^4^. ICIs alone or in combination with other cancer treatment options have achieved significant success as a standard treatment in several cancer indications^8–11^. To date, the FDA has approved seven commercial ICIs^12^. However, due to the burgeoning cost of these cancer treatments, patients have limited access to them^13,14^.

Recombinant proteins for human use are prohibitively expensive due to the high cost of manufacturing. When compared to other production platforms, the plant platform has many advantages, including faster production in the case of transient expression^15^, scalability^16^, lower upstream production costs than mammalian cells^17,18^, and a lower risk of human pathogen contamination^19^. Plants are also capable of posttranslational modifications, which are required for complex proteins like mAbs^20^. Previous research demonstrated the capabilities of plant platform in producing recombinant mAbs against Ebola^21^, rabies^22^, and oncology applications^23–25^.

In this study, the plant platform was used to produce anti-PD-L1 mAb and determine its activity. The purified plant-produced Atezolizumab was characterized using SDS-PAGE and western blot and its activity was compared with the commercial anti-PD-L1 mAb (Tecentriq). Results showed that the plant-produced Atezolizumab was slightly larger in size than Tecentriq. In terms of functional analysis, the plant-produced Atezolizumab demonstrated similar results in binding to huPD-L1 *in vitro* and reducing tumor weight and volume in mice *in vivo*. Our data confirms that the plant system can produce biologically active proteins with functions similar to those of other well-established platforms. More importantly, this platform has the potential to reduce associated costs in the upstream process of drug production, thereby increasing patient access to biological treatments.

## Results

### Expression of recombinant atezolizumab in *N. benthamiana*

**Figure 1:**
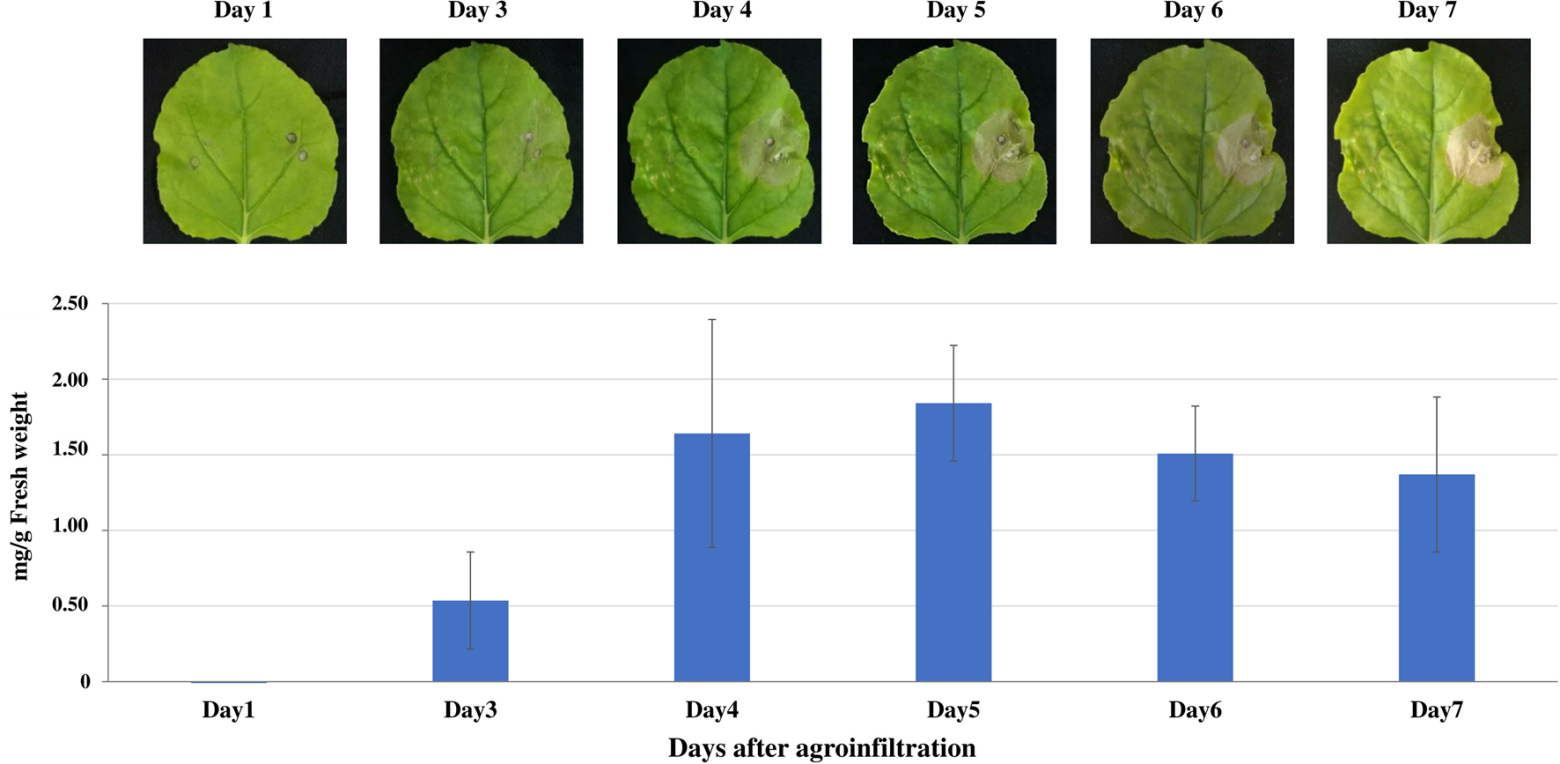
Day optimization experiment for plant-produced Atezolizumab. Infiltrated *N. benthamiana* leaves were harvested on days 1, 3, 4, 5, 6, and 7 post-infiltration (dpi). The antibody expression level at various dpi was calculated by sandwich ELISA. Representative images of leaf necrosis and a graph showing the relative expression of plant-produced Atezolizumab were provided. Data are presented as mean ± SD of triplicate samples.

To generate a non-glycan version of Atezolizumab, three amino acids (N298A, D359E, and L361M) of heavy chain were mutated using overlap PCR. The gene was inserted into a geminiviral vector and transformed into *A. tumefaciens*. The transformed bacterial cells containing anti-PD-L1 non-glycan heavy chain or light chain were co-infiltrated into *N. benthamiana* leaves. The level of protein expression was determined using day optimization. Accordingly, the infiltrated leaves were harvested at various days post infiltration (1, 3, 4, 5, 6 and 7 dpi) and the expression levels of Atezolizumab were measured by quantitative sandwich ELISA. The presence of symptoms on the infiltrated leaf area confirms the expression of mAb. However, when necrosis occurred on the later days, Atezolizumab expression decreased. The highest expression level of plant-produced Atezolizumab yielded approximately 1.8 mg/g fresh weight within 5 dpi (Fig. 1). SDS-PAGE and western blot were used to compare infiltrated crude extract to non-infiltrated crude extract (Supplementary Figs. 1 and 2). Under reducing and non-reducing conditions, the crude proteins were stained by InstantBlue dye (Supplementary Fig. 1a) and the expression of Atezolizumab in infiltrated *N. benthamiana* extract revealed bands at 50 and 150 kDa using anti-human IgG (Supplementary Fig. 1b, lane 2) and at 25 and 150 kDa using anti-human Kappa (Supplementary Fig. 1c, lane 2), respectively. As expected, these bands were absent in non-infiltrated *N. benthamiana* extract (Supplementary Figs. 1b,c, lane 1).

### Purification of plant-produced atezolizumab from *N. benthamiana* proteins

**Figure 2:**
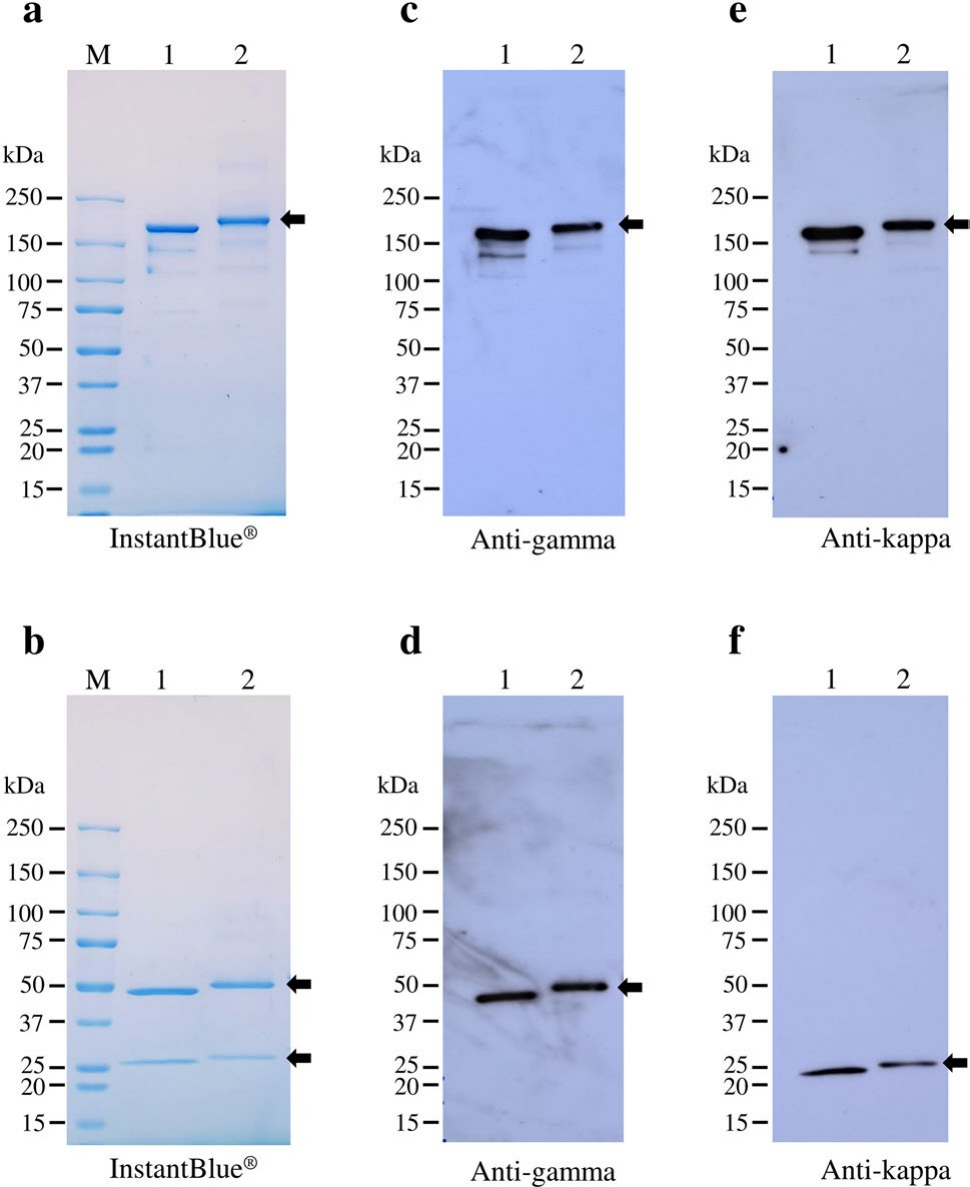
SDS-PAGE and western blot analysis of anti-PD-L1 mAbs. Tecentriq and purified plant-produced Atezolizumab were separated by non-reducing (**a**, **c**, and **e**) and reducing SDS-PAGE (**b**, **d**, **f**). About 2 µg of mAbs were loaded in SDS-PAGE, and 300 ng of mAbs were loaded for western blot analysis. The gels were either stained with InstantBlue dye (**a**,**b**) or transferred to nitrocellulose membrane and probed with anti-human IgG (heavy chain-specific) (**c**,**d**) and anti-human kappa (light chain-specific) (**e**,**f**). Lane M: protein ladder; lane 1: Tecentriq; lane 2: purified plant-produced Atezolizumab.

Plant-produced Atezolizumab was purified from infiltrated *N. benthamiana* crude extract by protein A affinity chromatography. The characteristics of purified plant-produced Atezolizumab was examined by SDS-PAGE and western blot (Fig. 2 and Supplementary Fig. 3). Under non-reducing condition, the plant-produced Atezolizumab was detected at 150 kDa with InstantBlue (Fig. 2a), anti-human IgG (Fig. 2c), and anti-human Kappa (Fig. 2e). Under reducing condition, the plant-produced Atezolizumab was observed at 50 kDa of heavy chain and 25 kDa of light chain with InstantBlue (Fig. 2b), anti-human IgG (Fig. 2d), and anti-human Kappa (Fig. 2f). On the other hand, the plant-produced Atezolizumab was slightly larger, with bands of higher molecular weight than Tecentriq (Fig. 2, lanes 1 and 2). The results of western blots probed with anti-human-IgG and anti-human Kappa confirm the co-expression of heavy and light chains, resulting in fully assembled mAb.

### Glycan analysis of plant-produced atezolizumab

**Figure 3:**
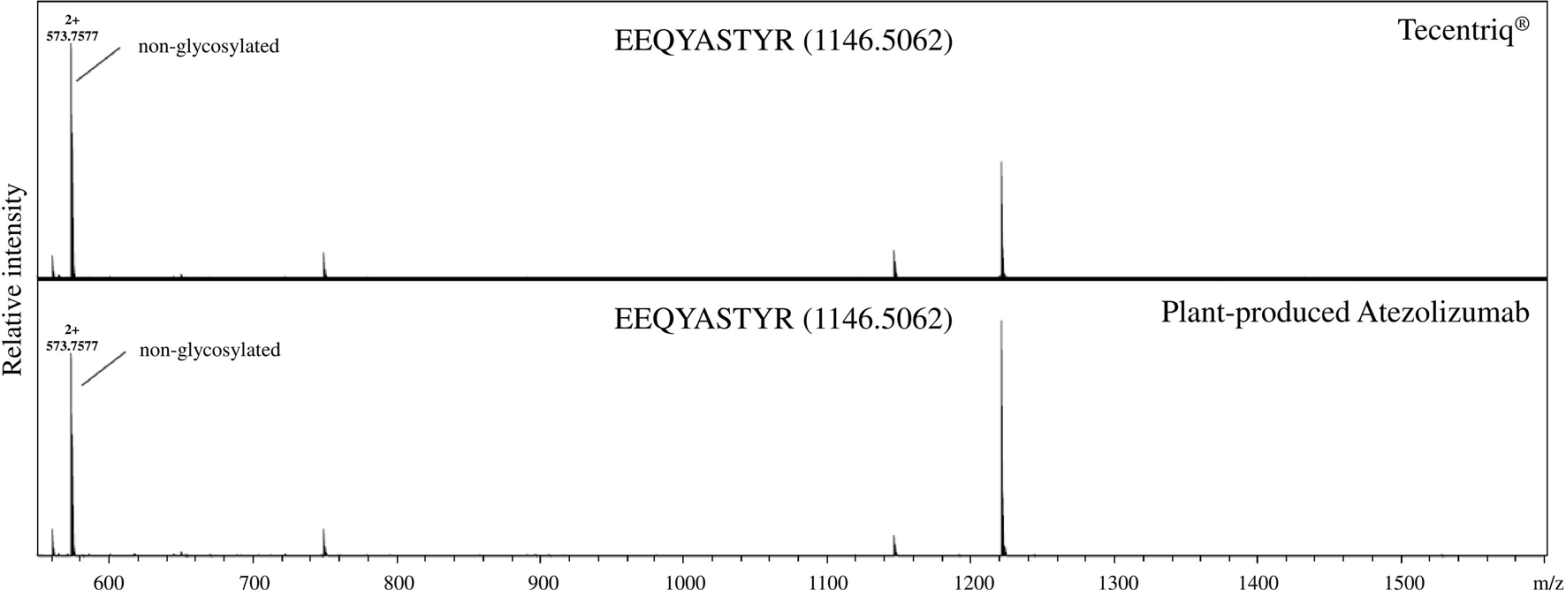
Glycosylation analysis of Tecentriq and purified plant-produced Atezolizumab. Tecentriq and purified plant-produced Atezolizumab were trypsin digested and analyzed with LC–ESI–MS.

The *N*-glycan composition of plant-produced Atezolizumab heavy chain was analyzed using liquid chromatography-electrospray ionization-mass spectrometry (LC-ESI-MS) analysis and compared to Tecentriq. Three sites in the mAb Fc region were mutated in an attempt to inhibit *N*-glycosylation of our plant-produced mAb. The findings confirmed that the mutant plant-produced Atezolizumab lacks glycan structures and follows the same pattern as Tecentriq (Fig. 3).

### Plant-produced atezolizumab binds to human PD-L1

**Figure 4:**
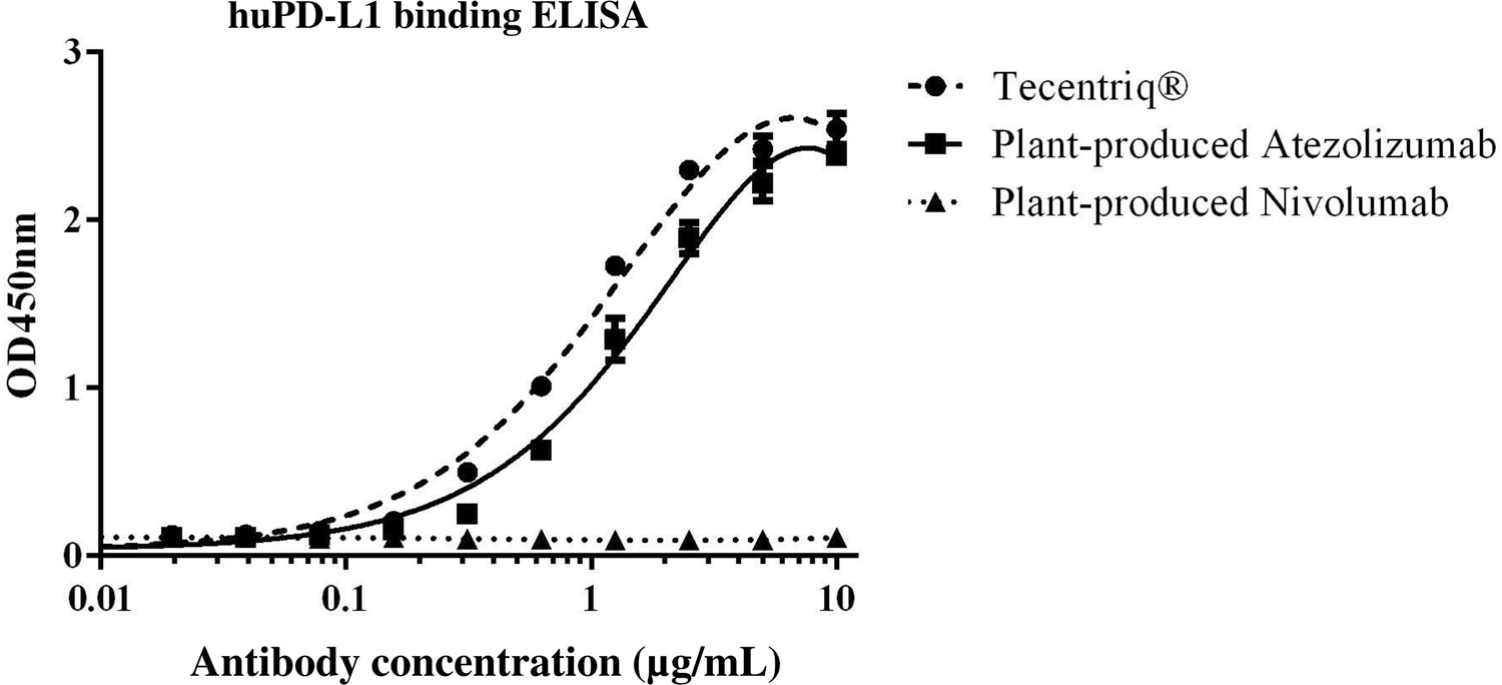
Binding affinity of purified plant-produced Atezolizumab to huPD-L1 protein at 2 µg/mL. Tecentriq was used as positive control and plant-produced Nivolumab as negative control. Increasing dilution series of mAbs were tested for huPD-L1 binding, which was detected by anti-human kappa-HRP. Data are presented as mean ± SD of triplicate samples.

The specific antigen recognition of plant-produced Atezolizumab was determined and compared to that of Tecentriq and plant-produced Nivolumab^23^ by functional ELISA. Both the plant-produced Atezolizumab and Tecentriq demonstrated specific and comparable binding to the huPD-L1 protein, whereas plant-produced Nivolumab showed no antigen binding (Fig. 4). This data confirmed that plant-produced Atezolizumab can bind to huPD-L1 *in vitro* and that triple mutations in the constant region of the heavy chain had no effect on the plant-derived mAb’s binding ability.

### Plant-produced atezolizumab elicits antitumor activity in mice

**Figure 5:**
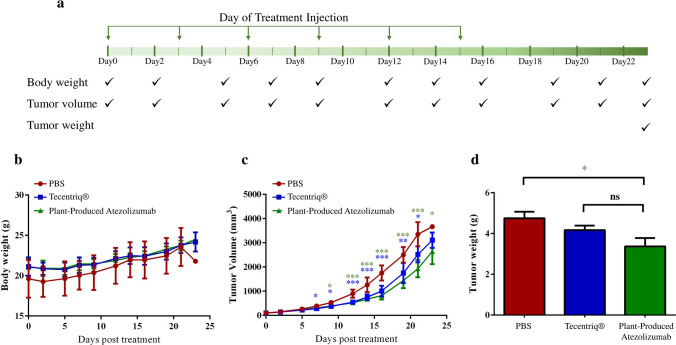
Antitumor efficacy of plant-produced Atezolizumab in CT26-hPD-L1-bearing knocked-in mice. Tecentriq was used as a positive control and PBS as a negative control. Three groups were formed (*n* = 6 mice/group) and injected with anti-PD-L1 mAbs (3 mg/kg i.p.) and PBS. The timeline for dose administration and data collection was illustrated in (**a**). Treatment-related effects were demonstrated on body weight (**b**), tumor volume (**c**), and tumor weight (**d**). Data were analyzed by using one-way ANOVA and presented as mean ± SD. *P < *0.05*, **P < *0.01*, ***P < *0.001* were considered as statistically significant.

To assess the tumor growth inhibitory (TGI) efficacy of plant-produced Atezolizumab, a BALB/c-hPD-1/hPD-L1/hCTLA-4 mouse model was subcutaneously transplanted with mouse colon tumor CT26 cells expressing hPD-L1. The plant-produced anti-PD-L1 mAb was administered to the mice along with Tecentriq as a positive control and PBS as a negative control, following the predetermined regimen in Fig. 5a. Mice were given 6 doses of each mAb drug at 3 mg/kg every three days, and tumor volumes (TV) and mouse body weight data were collected until Day 23. As shown in Fig. 5b, mice in all three treatment groups had similar body weights, indicating good tolerance to continuous treatment administration. On day 21, the plant-produced Atezolizumab (TGI_TV_ = 41.90%) and Tecentriq (TGI_TV_ = 24.59%) significantly reduced the growth of CT26-hPD-L1 tumors in mice when compared to the negative control (Supplementary tables 1 and 2) (*P*<0.05). Furthermore, tumor volume reduction did not differ between anti-PD-L1 mAb-treated groups (Fig. 5c) (*P* > 0.05). At the end of the study, all mice were terminated, and tumors were collected and weighed (tumor weight; TW). The tumor sizes in groups treated with plant-produced Atezolizumab (TGI_TW_= 29.03%) were significantly smaller than those in the PBS control group (*P* < 0.05), as depicted in Fig. 5d and Supplementary table 3. Most importantly, the antitumor efficacy of plant-produced Atezolizumab was not significantly different from that of its mammalian cell-produced mAb counterpart in this syngeneic murine colorectal cancer model (*P* > 0.05).

## Discussion

Immune checkpoint inhibitors (ICIs) are a type of immunotherapy that is increasingly relevant in cancer treatment. These anti-cancer agents have shown robust and long-term clinical benefits, but they are also expensive^14,26^. The lengthy timeline of research and development, financial obligations of clinical trials, and cost of production process are just few of the many factors that lead to the prohibitive pricing of biopharmaceutical medicines. Until now, the affordability of cancer care and biologics has always been a major concern. Drug prices should be reduced so that more patients, particularly those in underdeveloped nations, can receive adequate healthcare and cancer treatment. In doing so, the manufacturing cost of drugs can be lowered via maximizing production technologies. Plants represent a promising platform for the production of biopharmaceutical products at relatively low cost^17,27,28^. They have the potential to rapidly produce plant-made therapeutics en masse with low contamination risk^19^. The production and economic advantages of plants fueled its promise as a competent pharmaceutical factory. Plant cells, tissues, or whole plants are among the primary systems used in the manufacturing of therapeutic recombinant proteins for commercial, industrial, or pharmaceutical applications^29^. To investigate the plant platform, commercial Atezolizumab (Tecentriq) produced in mammalian cells, which is one of seven commercial ICIs approved by the FDA^12^, was used as a control in comparison to Atezolizumab produced in *N. benthamiana*.

In previous studies, geminiviral vectors have set the stage for transient expression of effective recombinant proteins and mAbs derived from plants^23,30,31^. Efforts to improve the protein expression levels in plants by exploiting targeting sequences have long been considered. The endoplasmic reticulum (ER) retention motif, SEKDEL (Ser-Glu-Lys-Asp-Glu-Leu), for example, demonstrated more efficient targeting, leading to enhanced accumulation of proteins in plants^32,33^. In addition, using cell secretion signals such as murine signal peptide has shown increased levels of plant-produced mAbs against rabies virus, whereby rabies mAb constructs carrying this murine signal sequence were more highly expressed than those carrying a plant signal peptide^34^. As demonstrated by our previous study, high level of glycosylated Atezolizumab expression was also found in *N. benthamiana* plants containing the heavy chain and light chain genes with a murine signal peptide on the N-terminus along with a SEKDEL sequence on the C-terminus^24^. Similarly, we have utilized these transgenes into this study and introduced mutations (N298A, D359E, and L361M) into the heavy chain, in order to alter the normal glycosylation of the mAb. Our results revealed that the mutated Atezolizumab was successfully expressed in plants, with highest amounts reaching up to 1.8 mg/g fresh weight in crude extracts within 5 days post-infiltration, which was consistent with prior report^24^. Generally, geminiviral-based vectors achieve transient expression of plant-made biopharmaceutical proteins in 2-6 days after agroinfiltration, depending on the plant leaf hypersensitive response^25,35,36^.

The plant-produced mAb was further purified and analyzed for physicochemical characteristics and molecular structures. Our findings show that heavy chain and light chain bands migrated closely to their expected molecular weights (50 and 25 kDa) under reducing SDS-PAGE and western blot. Likewise, non-reducing SDS-PAGE and immunoblotting analyses revealed that the plant-produced mutated Atezolizumab was completely and correctly assembled (150 kDa). However, the apparent molecular weights of the heavy chain, light chain, and assembled plant-synthesized mAb were slightly higher when compared with Tecentriq. This small increase in size may be attributed to the addition of SEKDEL^37–39^ and the 19-amino acid murine signal peptide^40^. Another possible explanation could be due to the unsuccessful cleavage of signal peptide^39^, which we have not confirmed. Asparagine (*N*)-linked glycosylation is a common post-translational modification involved in several biological functions such as protein folding, stability, biological activity, interaction, and others ^41,42^. The early stages of plant *N*-glycosylation appear to be conserved and similar to that of mammalian cells^43^, which usually occurs in the ER^44^. mAb *N*-glycan processing has become a crucial aspect in biologic manufacturing. In the case of commercial Atezolizumab (Tecentriq), glyco-modification via amino acid substitution at position 298 (asparagine to alanine; N298A) of the heavy chain results in a non-glycosylated mAb. This removal of *N*-glycans reduces or abrogates the binding affinity of anti-PD-L1 mAb for Fcγ receptors^45,46^. Herein, using our plant expression platform, we generated a non-glycosylated Atezolizumab by incorporating glycan-deleting mutations in its Fc region and compared it to Tecentriq. Site-specific glycosylation was analysed by LC-ESI-MS and no glycan structures were identified in our plant-produced Atezolizumab. These findings confirm the successful removal of *N*-glycans following mutations on the conserved glycosylation site and are consistent with the data from non-glycosylated reference mAb. Furthermore, the murine signal peptide and SEKDEL motif fused to plant-produced mAb only act as leading and targeting sequences for protein expression. However, it is well-known that aglycosylation of mAbs yields to aggregation^42^, which in turn may induce anti-drug antibodies^47^. More recently, several studies attempted to produce glycosylated variants of Atezolizumab in the hopes of improving antibody stability and activity^48,49^. Furthermore, two amino acid substitutions were introduced at positions 359 (glutamic acid to aspartic acid; D359E) and 361 (leucine to methionine; L361M) to follow the protein sequence of Tecentriq. These allotypic residues in IgG1 were altered with corresponding amino acids from IgG2, resulting in reduced mAb binding affinity to FcγR^50^.

The plant-produced Atezolizumab binds specifically to human PD-L1 protein in functional ELISA. It also exhibited comparable binding with Tecentriq, whereas plant-produced Nivolumab did not bind to the target as predicted. These findings indicate that Fc modifications had no effect on the antigen-binding capacity of our plant-produced mAb, in agreement with previous studies^24,48^. Overall, the results presented here illustrate that a functional non-glycosylated anti-PD-L1 mAb can be produced in plants with specific binding activity to PD-L1. Atezolizumab immunotherapy has been considered as a first-line treatment option for patients with metastatic lung cancer^51^ and some patients with advanced urothelial cancer ^52^. It has also been used in combination to treat certain types of cancer, including liver, breast, and colon cancer^53–55^. In this study, we evaluated the efficiency our plant-produced Atezolizumab in syngeneic mouse model subcutaneously grafted with murine CT26-hPD-L1 tumors. At a dose of 3 mg/kg, our findings revealed significant inhibition of tumor growth from plant-derived mAb treatment. Notably, the plant-produced Atezolizumab demonstrated similar regression of tumor volumes and weights as compared with Tecentriq. In addition, the antitumor responses in anti-PD-L1 mAb-treated groups differed significantly from those in PBS control group. Prior research has shown that high doses of Atezolizumab (10 mg/kg) increased antitumor activity *in vivo*^48^. Having said that, dose-escalation experiments for our plant-produced Atezolizumab may be investigated in the future to optimize tumor growth inhibitory response. On the other hand, tumor-bearing mice displayed no significant body weight changes in any of the treatment groups, indicating that plant-produced Atezolizumab treatment is safe and tolerable^56^.

In conclusion, we have successfully generated an anti-PD-L1 Atezolizumab in *N. bethamiana* plants with no glycosylation in its heavy chain. The glyco-modified plant-produced Atezolizumab had the same *N*-glycan pattern as the aglycosylated commercial Atezolizumab (Tecentriq). Moreover, both the plant-based mAb and Tecentriq had comparable antigen binding affinities to human PD-L1 *in vitro*. Most importantly, our plant-derived Atezolizumab is as effective as Tecentriq in inhibiting tumor growth *in vivo*. These findings confirm the feasibility of plant platforms both for biopharmaceutical production as well as for immunotherapy. If desired, plants could be considered as another production system of choice, with the goal of lowering the cost of biopharmaceutical drugs in developing countries.

## Methods

### Research involving plants

The study was permitted to be carried out by the internal CU-IBC (Chulalongkorn University-Institutional Biosafety Committee) following all the Biosafety guidelines for modern biotechnology. All methods were performed in accordance with the relevant guidelines/regulations/legistration. The seeds of *N. benthamiana* used in the present study were kindly gifted by Dr. Supaart Sirikantaramas, Faculty of Science, Chulalongkorn University.

### Approval for animal experiments

This study (Animal Protocol No. GPTAP20220128-4) was reviewed and approved by the Institutional Animal Care and Use Committee (IACUC) of GemPharmatech Co., Ltd. (Nanjing, China). The care and use of animals for experiments were performed in accordance with the Animal welfare act and the Association for Assessment and Accreditation of Laboratory Animal Care (AAALAC). The study adheres to the recommendations in the ARRIVE guidelines.

### Gene cloning in expression vector

The heavy chain (HC) and light chain (LC) genes of Atezolizumab carrying an N-terminal murine signal peptide and a C-terminal SEKDEL peptide had previously been obtained^24^. Briefly, the gene encoding sequences for the HC and LC were optimized *in silico* with *N. benthamiana* codons by Invitrogen GeneArt Gene Synthesis (Thermo Scientifc, USA). The variable domains of Atezolizumab HC and LC were commercially synthesized and fused separately to the constant domains of human IgG1 gamma chain or kappa chain, respectively. Here, multiple nucleotide substitutions were incorporated into the coding sequence of HC by overlapping PCR method (N298A, D359E, and L361M) to generate a non-glycosylated Atezolizumab (NGAte) with desired mutations. The primers used to introduce specific point mutations into Atezolizumab HC are listed in Table 1. The resulting mutated HC product was confirmed by sequencing. Both the Atezolizumab HC and LC constructs were digested with *Xba*I and *Sac*I restriction enzymes (BioLabs, Massachusetts, USA) and subcloned into the pBY2eK geminiviral vector (provided by Professor Hugh Mason^30^). The *Agrobacterium tumefaciens* strain GV3101 was used for bacterial transformation.

**Table 1:** Primer sequences used for non-glycosylated Atezolizumab heavy chain creation.

Primer name	Sequencing (5’-3’)
XbaI-SP_F SacI-SEKDEL_R N-A_F N-A_R DEL-EEM_F DEL-EEM_R	GCTCTAGAACAATGGGCTGG CGAGCTCTCAAAGCTCATCCTTCTCAGA GAGAGAGGAACAGTACGCCAGCACGTACAGGGTTG CAACCCTGTACGTGCTGGCGTACTGTTCCTCTCTC CCTCCATCTCGCGAGGAAATGACCAAGAACCAGG CCTGGTTCTTGGTCATTTCCTCGCGAGATGGAGG

The *A. tumefaciens* cells containing pBY2eK-NGAte-HC and pBY2eK-Ate-LC were cultured in Luria Bertani (LB) media (HiMedia Laboratories, Mumbai, India) supplemented with kanamycin (50 mg/L), gentamycin (50 mg/L) purchased from AppliChem, Dermstadt, Germany, and rifampicin (25 mg/L) purchased from TOKU-E, Washington, USA. The bacterial cultures were grown overnight at 28°C with continuous shaking (200 rpm) and diluted to an OD_600_ of 0.2 in infiltration buffer (10 mM 2-(N-morpholino) ethanesulfonic acid (MES) pH 5.5, 10 mM MgSO_4_). Bacteria were then mixed in a 1:1 ratio and co-agroinfiltrated into 6–8-week-old *N. benthamiana* plants via syringe infiltration (small-scale) or vacuum infiltration (large-scale). The infiltrated plants were incubated in a controlled plant room and harvested on days 1, 3, 4, 5, 6, and 7 after agroinfiltration to examine time-dependent mAb expression levels. Leaves were extracted with 1×PBS and then centrifuged at 24,000×g for 15 min. mAb concentrations in crude samples were quantified by assaying the supernatant in sandwich ELISA. For purification experiments performed here, infiltrated plants were collected at the optimal harvest time of peak mAb production and homogenized. The plant-produced Atezolizumab was purified by protein A affinity chromatography, as described previously ^23^.

### SDS-PAGE and western blot analysis

SDS-PAGE and western blot assays were carried out to confirm the size and assembly of plant-made anti-PD-L1 mAb. Protein samples including non-infiltrated crude extract, agroinfiltrated crude extract, purified plant-produced Atezolizumab, and Tecentriq (Roche, Switzerland) were either mixed with non-reducing loading dye (125 mM Tris-HCl pH 6.8, 12% (w/v) SDS, 10% (v/v) glycerol, 0.001% (w/v) bromophenol blue) or reducing loading dye (non-reducing loading dye with 22% (v/v) β-mercaptoethanol). The samples were separated in 4-15% polyacrylamide gel and visualized by InstantBlue (Abcam, Cambridge, UK). For western blot analysis, the proteins were transferred to nitrocellulose membrane (Bio-Rad, Massachusetts, USA). The membrane was blocked with 5% (w/v) skim milk in 1×PBS. Goat anti-human Kappa-HRP (SouthernBiotech, Alabama, USA) at 1:5,000 and goat anti-human IgG-HRP (SouthernBiotech, Alabama, USA) at 1:10,000 in 3% (w/v) skim milk were used as detection antibodies. Blots were developed using ECL substrate (Promega, Wisconsin, USA) and exposed to X-ray film (Carestream, New York, USA).

### Antibody quantification

Concentration of mAb in crude and purified samples were calculated by quantitative sandwich ELISA. A 96-well, half-area microtiter plate (Corning, New York, USA) was coated with goat anti-human IgG Fc (Abcam, Cambridge, UK) overnight at 1:1,000 dilution in 1×PBS. Plate washing was with 1×PBS-T (1×PBS with 0.05% Tween 20), and blocking was with 5% (w/v) skim milk in 1×PBS. Serially diluted plant samples and human IgG1 kappa isotype antibody standard (Abcam, Cambridge, UK) were incubated on the coated plates for 2 h at 37 °C, followed by detection with goat anti-human Kappa-HRP (SouthernBiotech, Alabama, USA) at 1:3,000 in 1×PBS. Finally, TMB one solution substrate (Promega, Wisconsin, USA) was added and the reaction was quenched with 1M H_2_SO_4_. The absorbance was measured at 450 nm on a NS-100 Nano Scan microplate reader (Hercuvan, Shah Alam. Malaysia).

### *N*-Glycan analysis

Plant-produced Atezolizumab and Tecentriq were separated in reducing SDS-PAGE. The heavy chain band was excised, S-alkylated, and trypsin digested. The digested peptide was then analyzed by liquid chromatography-electrospray ionization-mass spectrometry (LC-ESI-MS), as described previously^57^.

### Binding assay to PD-L1

The binding ability of plant-produced Atezolizumab to human PD-L1 was investigated in a functional antigen-binding ELISA. A 96-well, half-area microtiter plate (Corning, New York, USA) was coated with recombinant huPD-L1 protein (R&D System, Minneapolis, USA) overnight at 2 µg/ml in 1×PBS. Then, the plate was washed with 1×PBS-T and blocked with 5% (w/v) skim milk in 1×PBS. Plant-produced Atezolizumab, Tecentriq standard, and plant-produced Nivolumab control^23^ were serially diluted and incubated on the coated plate for 2 h at 37 °C. Detection was with goat anti-human Kappa-HRP (SouthernBiotech, Alabama, USA) at 1:3,000 in 1×PBS and peroxidase activity was determined by TMB one solution substrate (Promega, Wisconsin, USA). Color development was monitored and stopped with 1M H_2_SO_4_. The absorbance was measured at 450 nm on a NS-100 Nano Scan microplate reader (Hercuvan, Shah Alam. Malaysia).

### Antitumor mouse model

Knocked-in female mice (strain BALB/c-hPD-1/hPD-L1/hCTLA-4) were provided and developed by GemPharmatech Co., Ltd, with experimental animal using license (SYXK (SU) 2018-0027) and experimental animal production license (SCXK (SU) 2018-0008). Briefly, the coding sequences of extracellular regions of PD-1, PDL-1 and CTLA-4 were replaced with human counterparts by CRISPR/Cas9 technology on BALB/c background (Supplementary Figs. 4–6). The expression of hPD-1, hPDL-1 and hCTLA-4 can be detected in this strain (Supplementary Fig. 7). Moreover, no obvious difference of T/B/NK cell proportion was observed between wild type and the humanized mice (Supplementary Fig. 8). Thus, this strain can be used to evaluate anti-tumor drugs.

The CT26 murine colon cancer cell line was procured from ATCC (Virginia, USA). The CT26-hPDL-1 cell line was developed by knocking-out mouse PD-L1 gene using CRISPR/Cas9 technology and inserting a constitutively expressed human PD-L1 gene. The cells were grown in Roswell Park Memorial Institute media (RPMI 1640, Gibco, NY, USA), supplemented with supplemented with 10% fetal bovine serum (ExCell Bio, Shanghai, China), 0.1% Penicillin/Streptomycin (Amresco, Ohio, USA) antibiotics, and 200 μg/mL G418 (Gibco, New York, USA). The mycoplasma-free cells were thawed and cultured at 37ºC and 5% CO_2_. Then, CT26-hPDL-1 cells (Passage: Pn+12) were collected and resuspended in Dulbecco's Phosphate-Buffered Saline (DPBS; Gibco, NY, USA). The cell viability was calculated before and after inoculation and found to be 97.14% and 95.17%, respectively. In this study, each humanized mice at the age of 6-8 weeks were injected subcutaneously with 1.10^6^ CT26-hPD-L1 cells into the right flank. All mice (*n*=18) were randomly separated into groups (*n*=6 mice/group) when tumor reached a diameter of 100 mm^3^. Treatment groups received either plant-produced Atezolizumab (3 mg/kg) or Tecentriq (3 mg/kg). The regimen consists of intraperitoneal (i.p.) antibody drugs every 3 days for up to 6 doses (Q3D×6). Mice from the vehicle group were injected with 1×PBS. The effect of treatment was determined by twice-weekly monitoring of tumor volume and mouse body weight. All mice were terminated on Day 23. At this time, tumors were collected for further analysis. Data are expressed as mean ± standard deviation (Mean ± SD) and analyzed using a one-way ANOVA test.

### Supplementary Information

Supplementary Information.

## Data Availability

All data generated or analysed during this study are included in this published article as supplementary information.
