# Supplementary material for: In vitro and in vivo studies of plant-produced Atezolizumab as a potential immunotherapeutic antibody
Source: Sci Rep. 2023 Aug 29;13:14146. doi: 10.1038/s41598-023-41510-w (PMC10465495; doi:10.1038/s41598-023-41510-w)
Supplement: Supplementary file 1 — Supplementary Information. [file 41598_2023_41510_MOESM1_ESM.docx]

In vitro and in vivo studies of plant-produced Atezolizumab as a potential immunotherapeutic antibody

# Kaewta Rattanapisit1, Christine Joy I. Bulaon2,3, Richard Strasser4, Hongyan Sun5, and Waranyoo Phoolcharoen2,3,*

Supplementary information

**
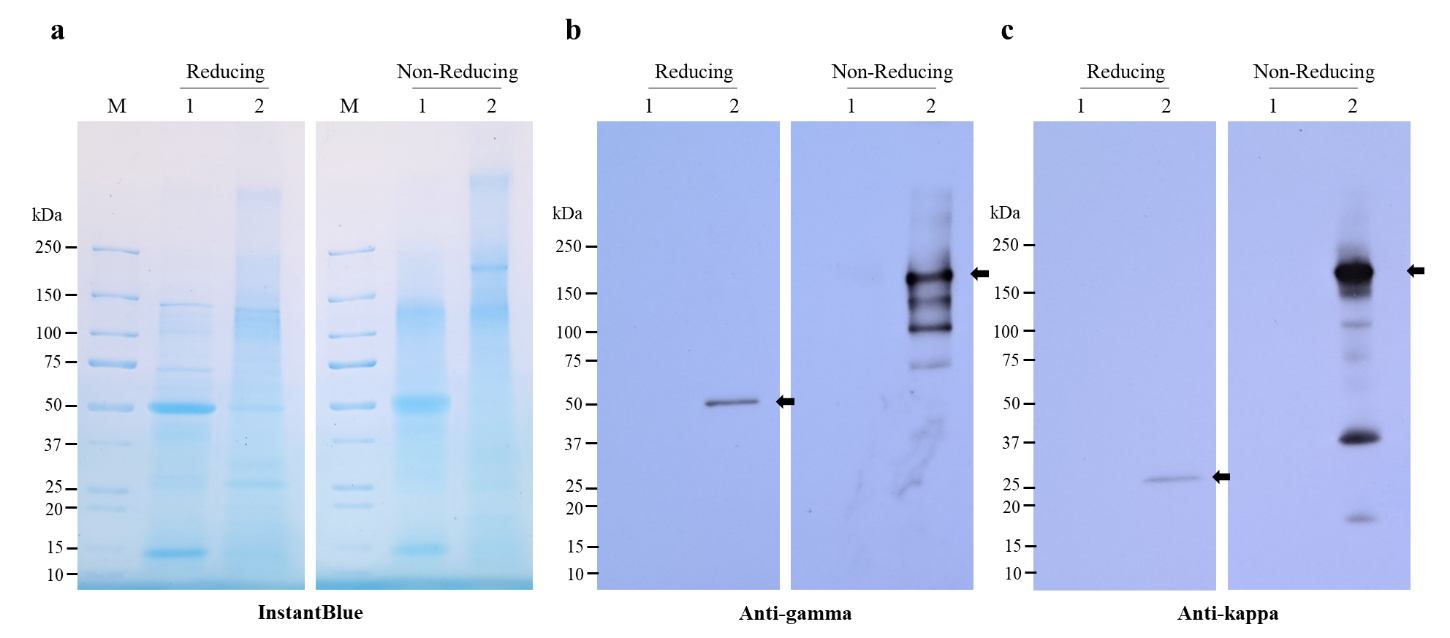
**

**Supplementary figure S1.** Expression of recombinant Atezolizumab in *Nicotiana benthamiana*. SDS-PAGE and chemiluminescence immunoblotting of anti-PD-L1 expression in crude leaf extracts (20 µg total soluble protein for SDS-PAGE and 2 ug total soluble protein for western blot) were analyzed under reducing and non-reducing condition. The SDS-PAGE gel was either stained with InstantBlue dye (A) or transferred to nitrocellulose membrane and probed with anti-human IgG (heavy chain-specific) (B) and anti-human kappa (light chain-specific) (C). Lane M: protein ladder; lane 1: crude proteins from non-infiltrated *N. benthamiana* leaves; lane 2: crude proteins from infiltrated *N. benthamiana* leaves.


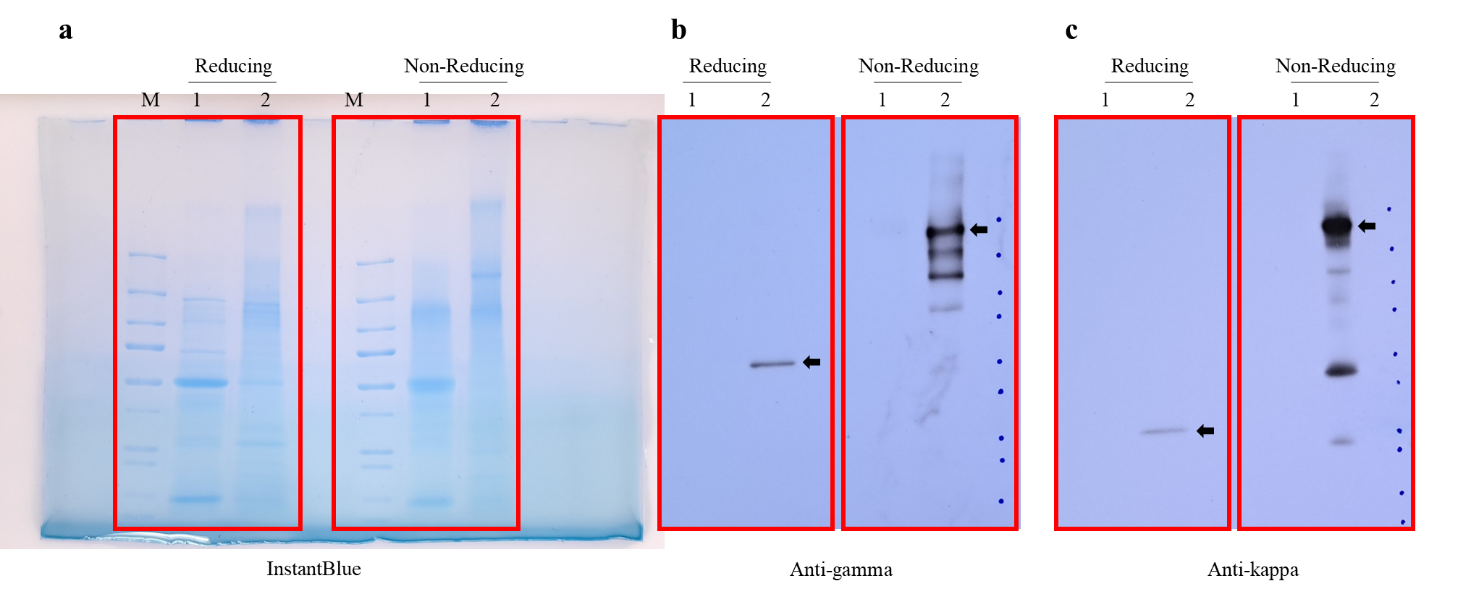


**Supplementary figure 2.** Raw gel and blot images related to Supplementary figure 1. Blot membranes with visible edges and of adequate length are not available due to confidentiality concerns.


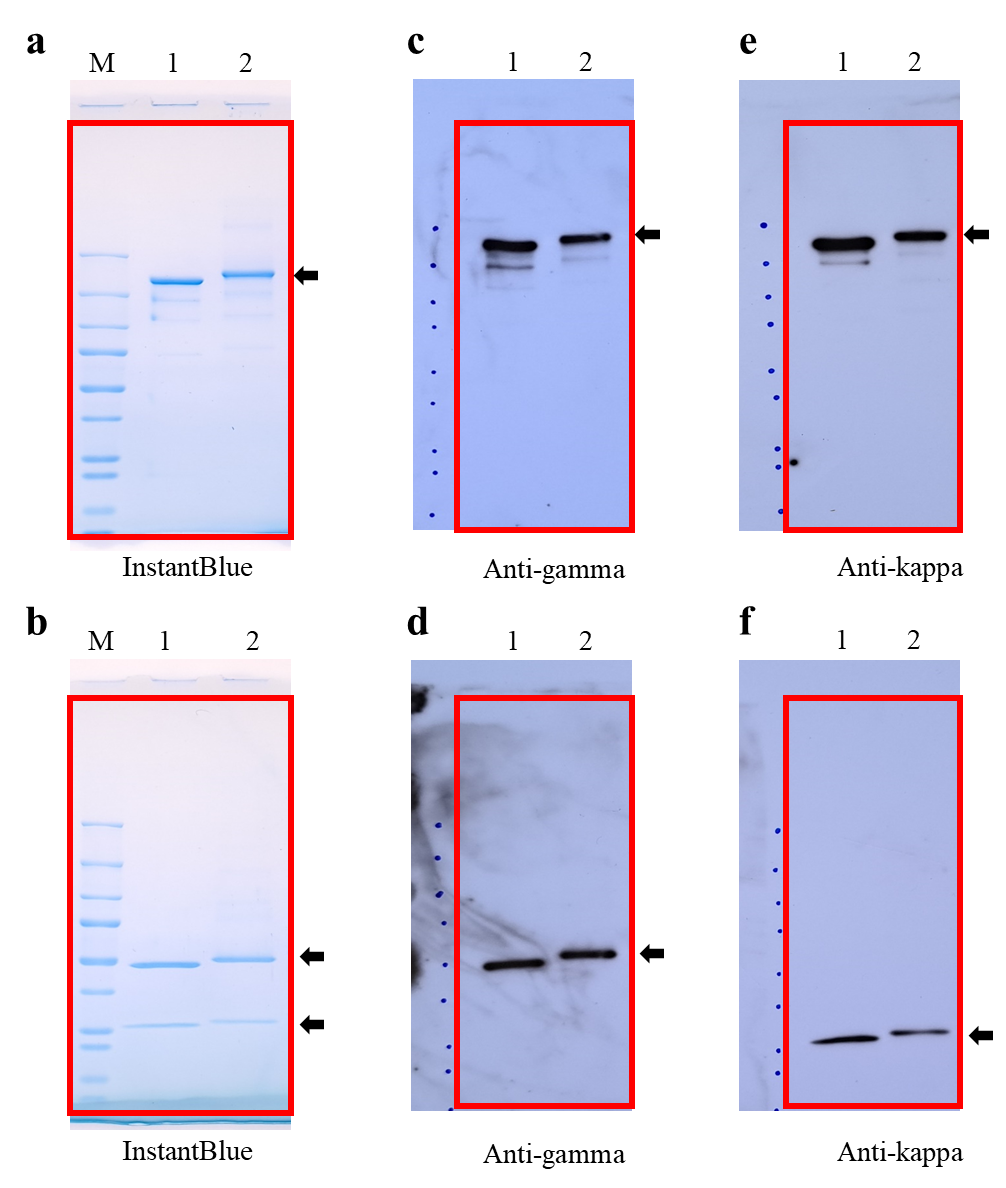


**Supplementary figure 3.** Raw gel and blot images related to Figure 2. Gels and blot membranes with visible edges and of adequate length are not available due to confidentiality concerns.


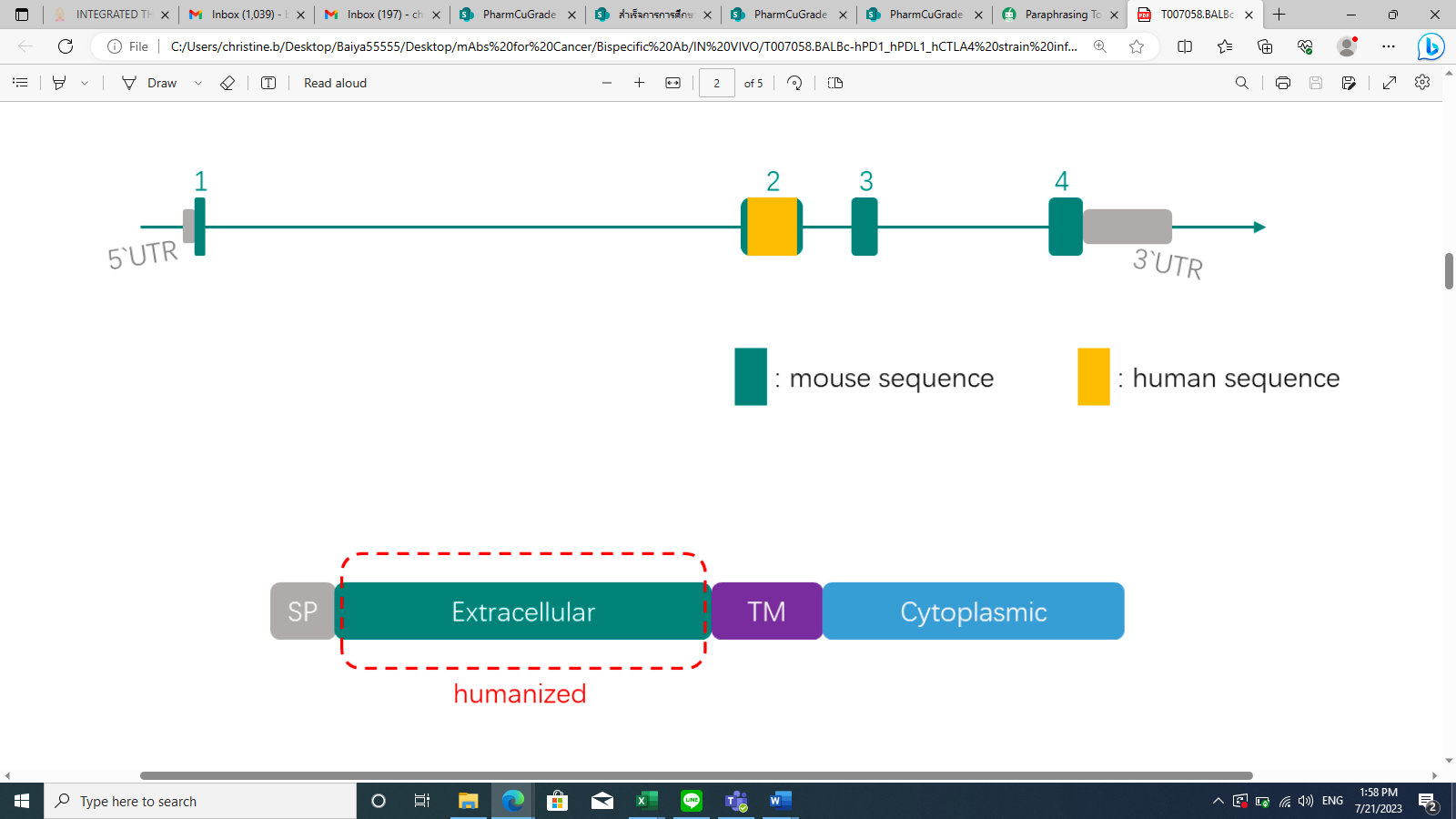


**Supplementary figure 4.** Schematic diagram of CTLA-4 humanization strategy in BALB/c-hPD-1/hPD-L1/hCTLA-4 mice.


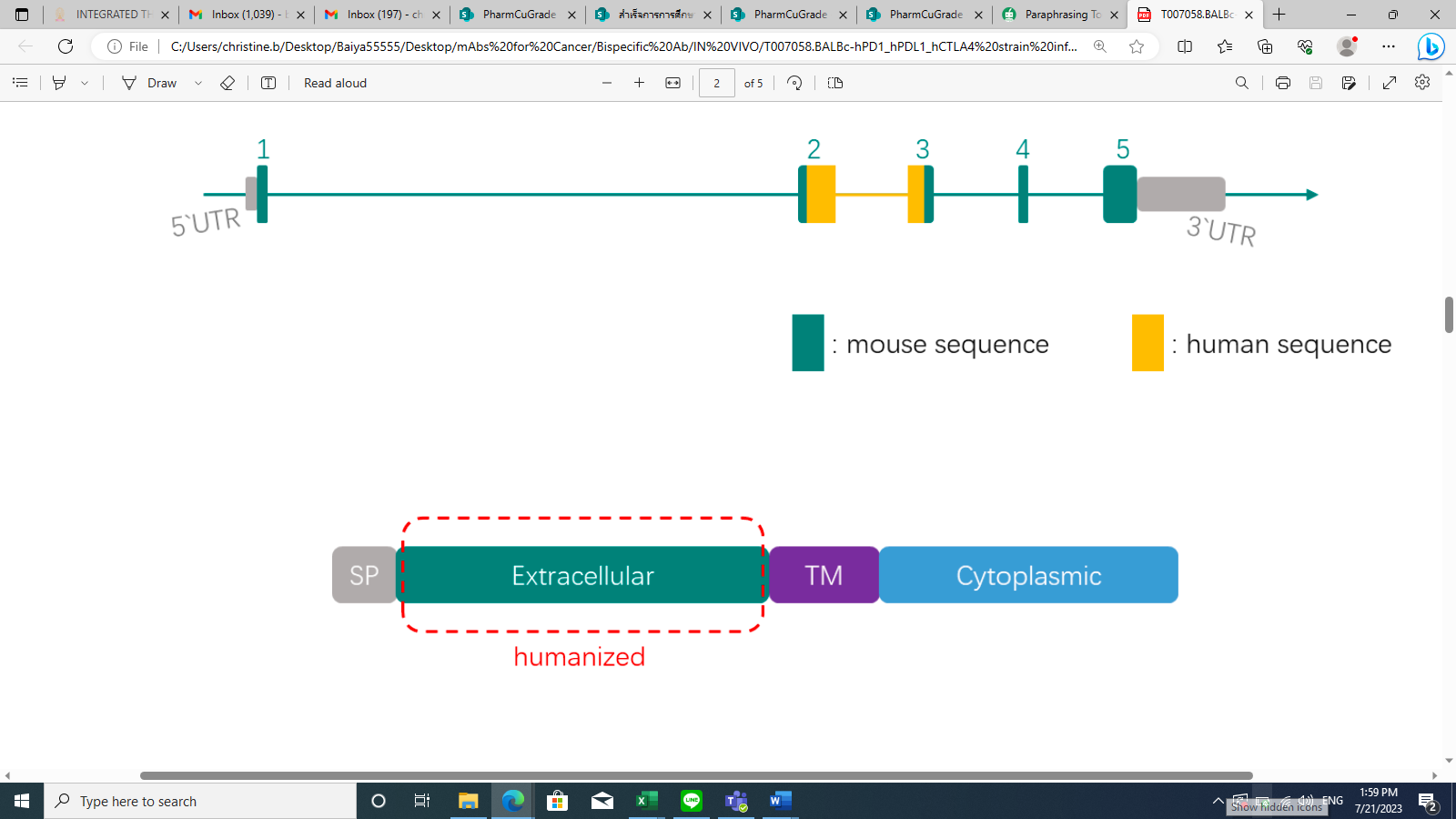


**Supplementary figure 5.** Schematic diagram of PD-1 humanization strategy in BALB/c-hPD-1/hPD-L1/hCTLA-4 mice.


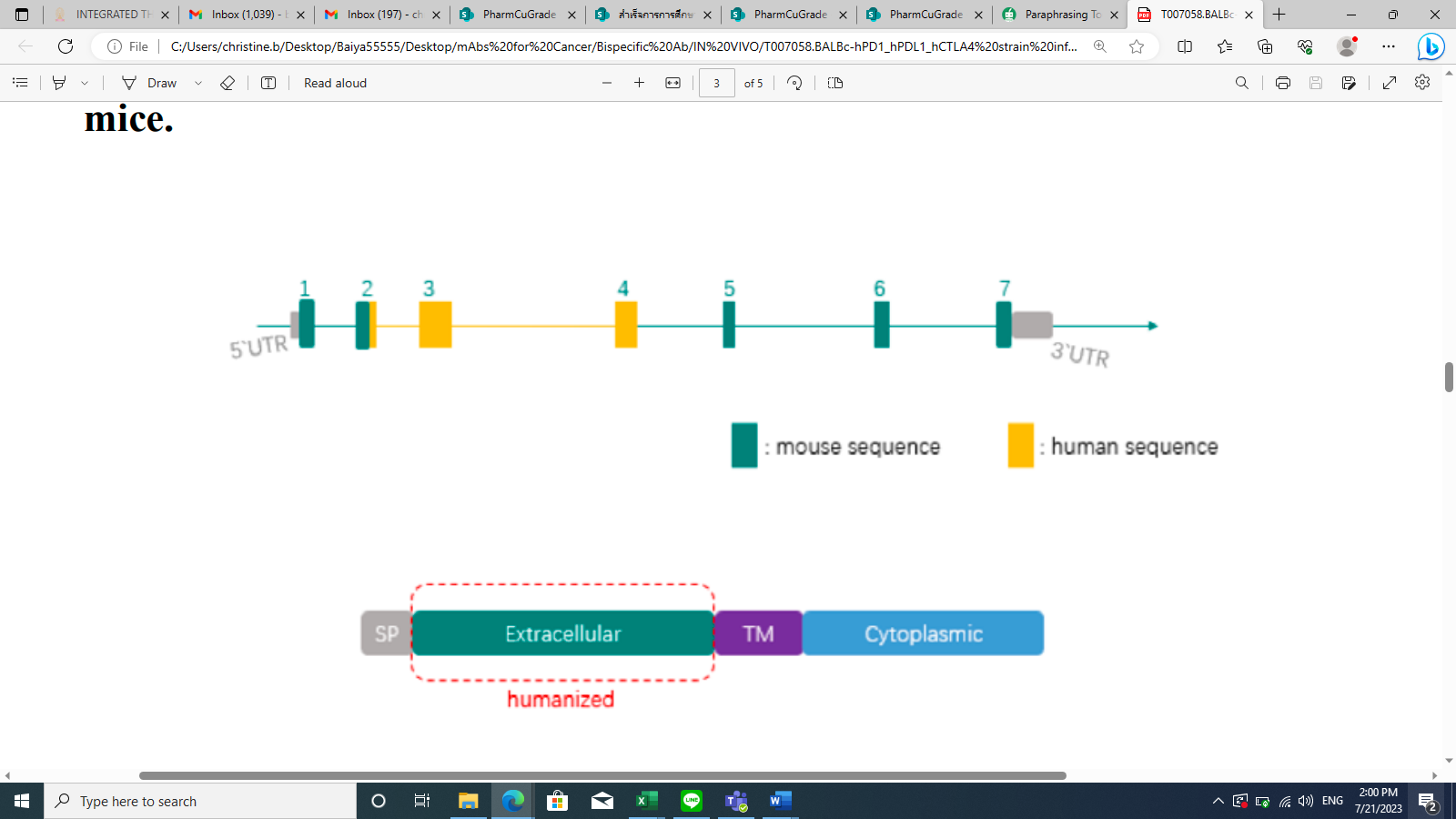


**Supplementary figure 6.** Schematic diagram of PD-L1 humanization strategy in BALB/c-hPD-1/hPD-L1/hCTLA-4 mice.


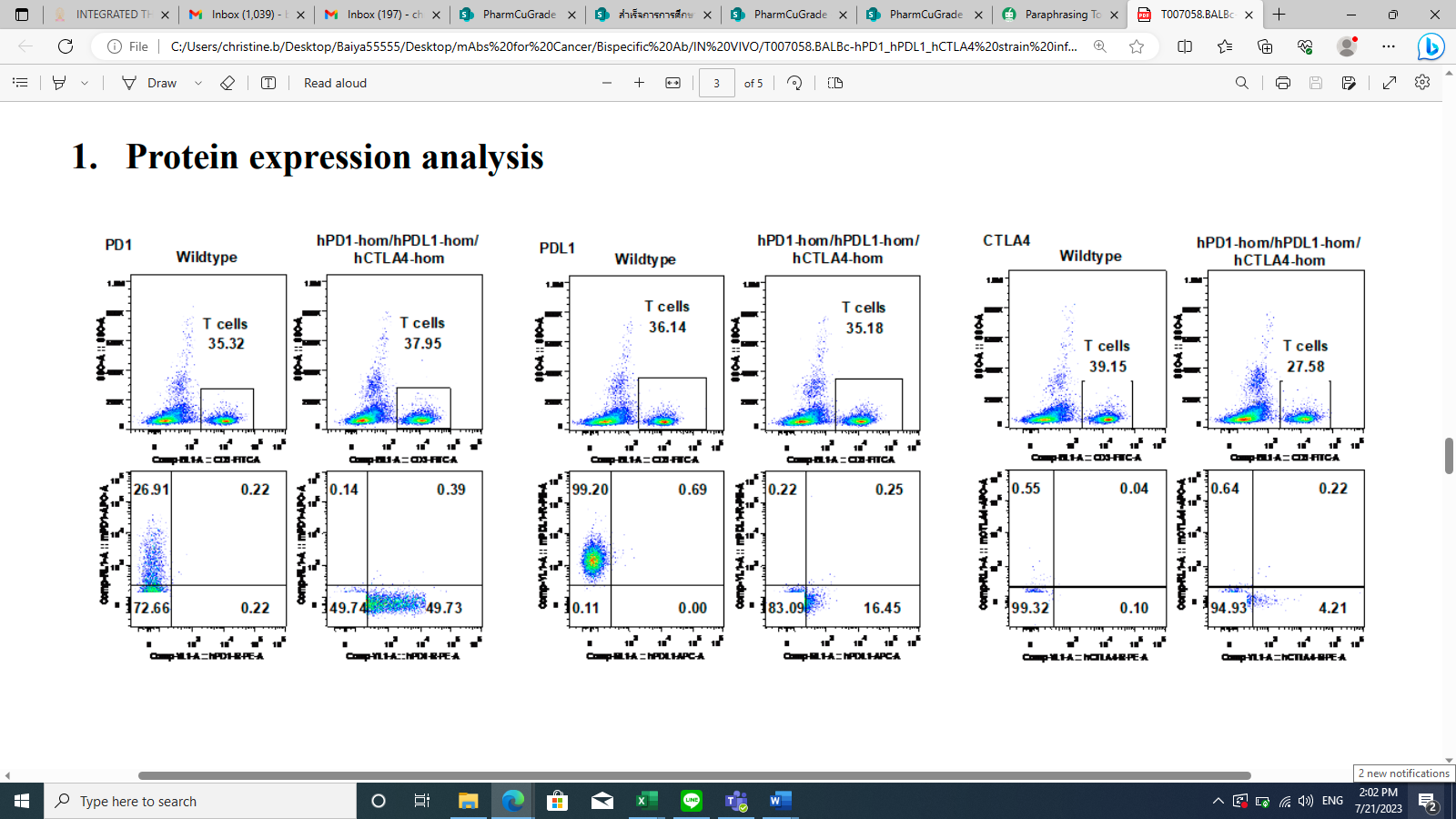


**Supplementary figure 7.** Detection of hCTLA-4, hPD-1, and hPD-L1 expression in BALB/c/hPD-1/hPD-L1/hCTLA-4 mice.


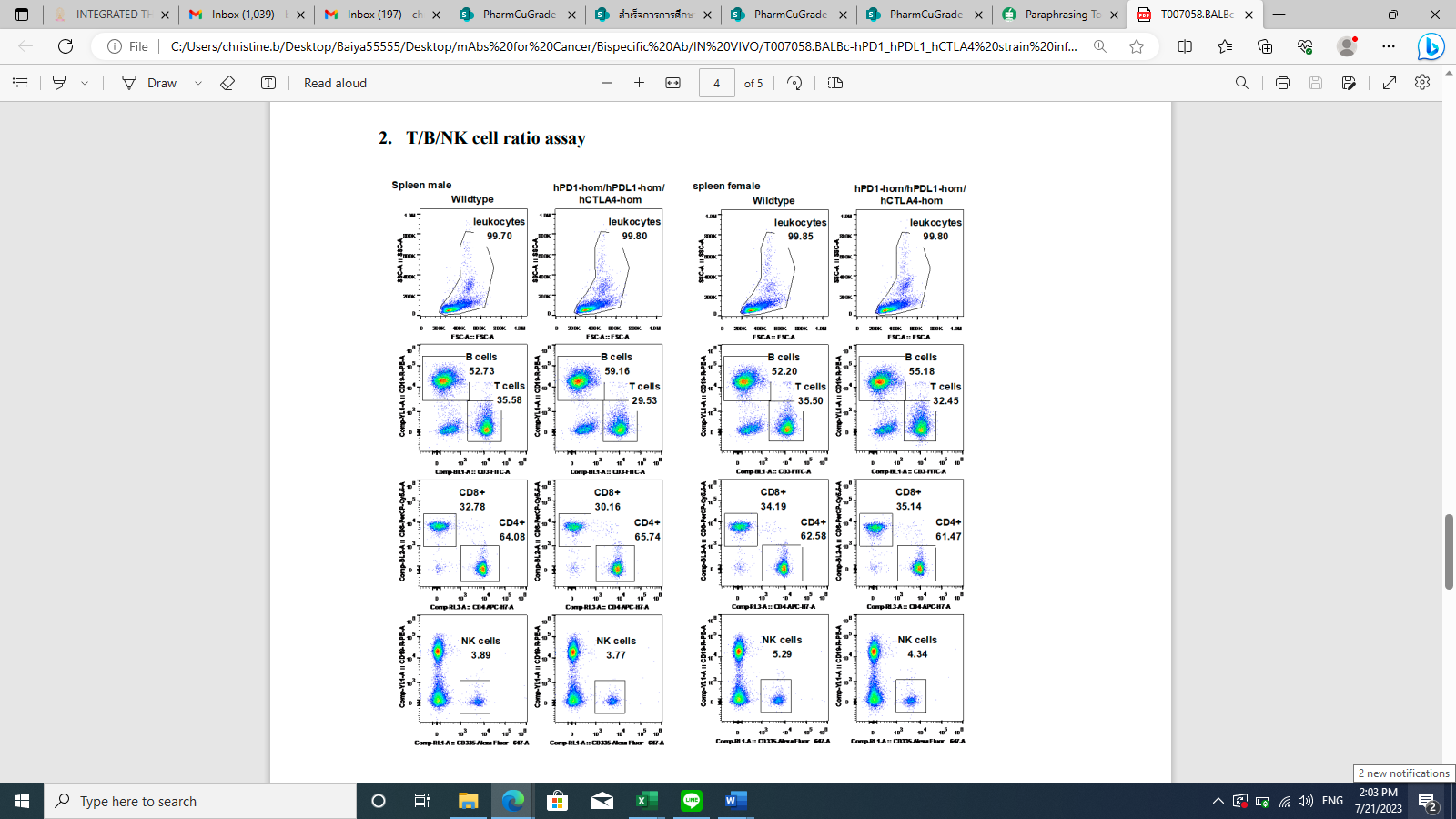


**Supplementary figure 8.** Detection of T/B/NK cell proportion in BALB/c/hPD-1/hPD-L1/hCTLA-4 mice.

**Supplementary table 1**

**Tumor Volume in different groups**

| **Group** | **Tumor Volume (mm^3^)** | | | | | | | | | | |
| --- | --- | --- | --- | --- | --- | --- | --- | --- | --- | --- | --- |
|  | **D0** | **D2** | **D5** | **D7** | **D9** | **D12** | **D14** | **D16** | **D19** | **D21** | **D23** |
| PBS | 100.91±  4.86 | 150.14±  8.34 | 264.24±  13.72 | 389.62±  20.81 | 530.60±  39.67 | 900.21±  68.63 | 1264.90±  122.43 | 1751.93±  127.13 | 2500.99±  130.60 | 3352.97±  201.59 | 3666.94±  30.93 |
| Tecentriq | 99.80±  3.97 | 143.26±  5.03 | 221.39±  12.13 | 280.02±  12.21 | 374.83±  24.72 | 552.62±  32.20 | 756.78±  48.91 | 1009.56±  88.29 | 1751.07±  171.11 | 2519.91±  135.12 | 3110.21±  129.51 |
| Plant-Atezolizumab | 99.82±  4.36 | 144.96±  11.72 | 236.12±  19.03 | 314.01±  32.03 | 388.87±  31.17 | 522.90±  27.27 | 672.55±  39.04 | 818.24±  63.67 | 1438.31±  127.79 | 1929.33±  144.88 | 2644.84±  214.53 |

Note: Data were shown as mean±SD.

**Supplementary table 2**

**TGI_TV_ Changes in different groups**

| **Group** | **D0** | **D2** | **D5** | **D7** | **D9** | **D12** | **D14** | **D16** | **D19** | **D21** | **D23** |
| --- | --- | --- | --- | --- | --- | --- | --- | --- | --- | --- | --- |
| PBS | - | - | - | - | - | - | - | - | - | - | - |
| Tecentriq | 0.00% | 3.27% | 16.10% | 28.09% | 29.45% | 38.51% | 40.06% | 42.62% | 29.63% | 24.59% | 10.86% |
| Plant-Atezolizumab | 0.00% | 2.19% | 10.15% | 18.70% | 25.99% | 41.23% | 46.41% | 53.16% | 41.65% | 41.90% | 23.99% |

Note: The calculation of TGI_TV_ was based on G1.

**Supplementary table 3**

**Statistical Analysis of Tumor Weight in different groups**

| **Group** | **Tumor weight (g)** | **TGI_TW_** | ***P* value**  **(vs PBS)** | ***P* value**  **(vs Tecentriq)** |
| --- | --- | --- | --- | --- |
| PBS | 4.7548 ± 0.3183 | - | - | - |
| Tecentriq | 4.1704 ± 0.2159 | 12.29% | 0.371 | 0.092 |
| Plant-Atezolizumab | 3.3746 ± 0.4031 | 29.03% | 0.041* | - |

Note: Data were shown as mean ± SD; Statistics performed using One-way ANOVA test, the post-hoc test was LSD, *: *P*<0.05, **: *P*<0.01, ***: *P*<0.001.
